# Chitosan-Coated Calcium Alginate Microspheres Loaded with Alpha-Lipoic Acid/2-HP-β-CD Inclusion Complexes Enhance Aqueous Solubility and Enable pH-Responsive Intestinal Release

**DOI:** 10.3390/ijms27208998

**Published:** 2026-10-11

**Authors:** Yu Cui, Shiya Huang, Yeming Fu, Zihao Zhou, Xiaowen Sun, Huadong Liu, Yuhong Lai

**Affiliations:** 1 School of Basic Medicine, Qingdao University, Qingdao 266071, China; cuiyu2@qdu.edu.cn; 2 NHC Key Laboratory of Cardiopulmonary Rehabilitation and Functional Recovery, School of Life Sciences and Health, University of Health and Rehabilitation Sciences, Qingdao 266113, China; huangshiya@uhrs.edu.cn (S.H.);; 3 School of Chemistry and Chemical Engineering, China University of Petroleum (East China), Qingdao 266580, China

**Keywords:** alpha-lipoic acid, 2-HP-β-CD, chitosan-coated calcium alginate microspheres, pH-responsive delivery, intestinal release

## Abstract

Alpha-lipoic acid (ALA) exhibits antioxidant and potential cardiovascular protective effects, but its insufficient stability in acidic environments and poor water solubility limit its oral delivery applications. In this study, the stability of lipoic acid in acidic environments was first evaluated through acid treatment combined with mass spectrometry analysis. Subsequently, 2-hydroxypropyl-β-cyclodextrin (2-HP-β-CD) and ALA were used to prepare lipoic acid inclusion complexes, which were characterized by phase solubility curves and X-ray diffraction (XRD). The inclusion complexes were further loaded into calcium alginate microspheres, and the properties of the microspheres were evaluated by scanning electron microscopy (SEM), morphological observation under different pH conditions, swelling ratio, and in vitro release experiments. Following acid treatment, the liquid chromatography–mass spectrometry (LC-MS) response of original ALA decreased, suggesting a reduction in detectable original ALA. The inclusion with 2-HP-β-CD significantly improved the solubility of lipoic acid and altered its crystalline characteristics. The inclusion complex-loaded calcium alginate microspheres exhibit a relatively intact microstructure, display distinct morphological changes and swelling behaviors under different pH conditions, and achieve pH-dependent release. This system may serve as a potential oral delivery platform for improving the aqueous dispersibility of ALA and promoting pH-responsive release under simulated intestinal conditions.

## 1. Introduction

Alpha-lipoic acid (ALA) is a naturally occurring sulfur-containing small molecule that functions as a cofactor for multi-enzyme complexes in mitochondrial energy metabolism [1], while also exhibiting various biological activities such as antioxidant, anti-inflammatory, redox homeostasis regulation, and improvement of metabolic abnormalities [2]. Due to the ability of ALA and its reduced form dihydrolipoic acid to participate in scavenging reactive oxygen species, regenerating endogenous antioxidants, and regulating cellular oxidative stress levels, it has shown potential application in diabetic neuropathy, inflammatory injury, metabolic disorders, and tissue protection [3]. In recent years, ALA has been widely used as a functional active ingredient in research on nutritional supplements and pharmaceutical preparations. However, its actual bioavailability remains significantly limited by its physicochemical properties and the gastrointestinal environment. Existing pharmacokinetic studies and reviews have indicated that ALA is rapidly absorbed after oral administration, yet it has a short half-life. Additionally, its oral bioavailability is typically only about 30–40%, influenced by factors such as poor water solubility, insufficient gastric stability, first-pass metabolism, and food intake [4]. These limitations have hampered the widespread application of ALA in clinical translation and scientific research.

**Figure 1:**
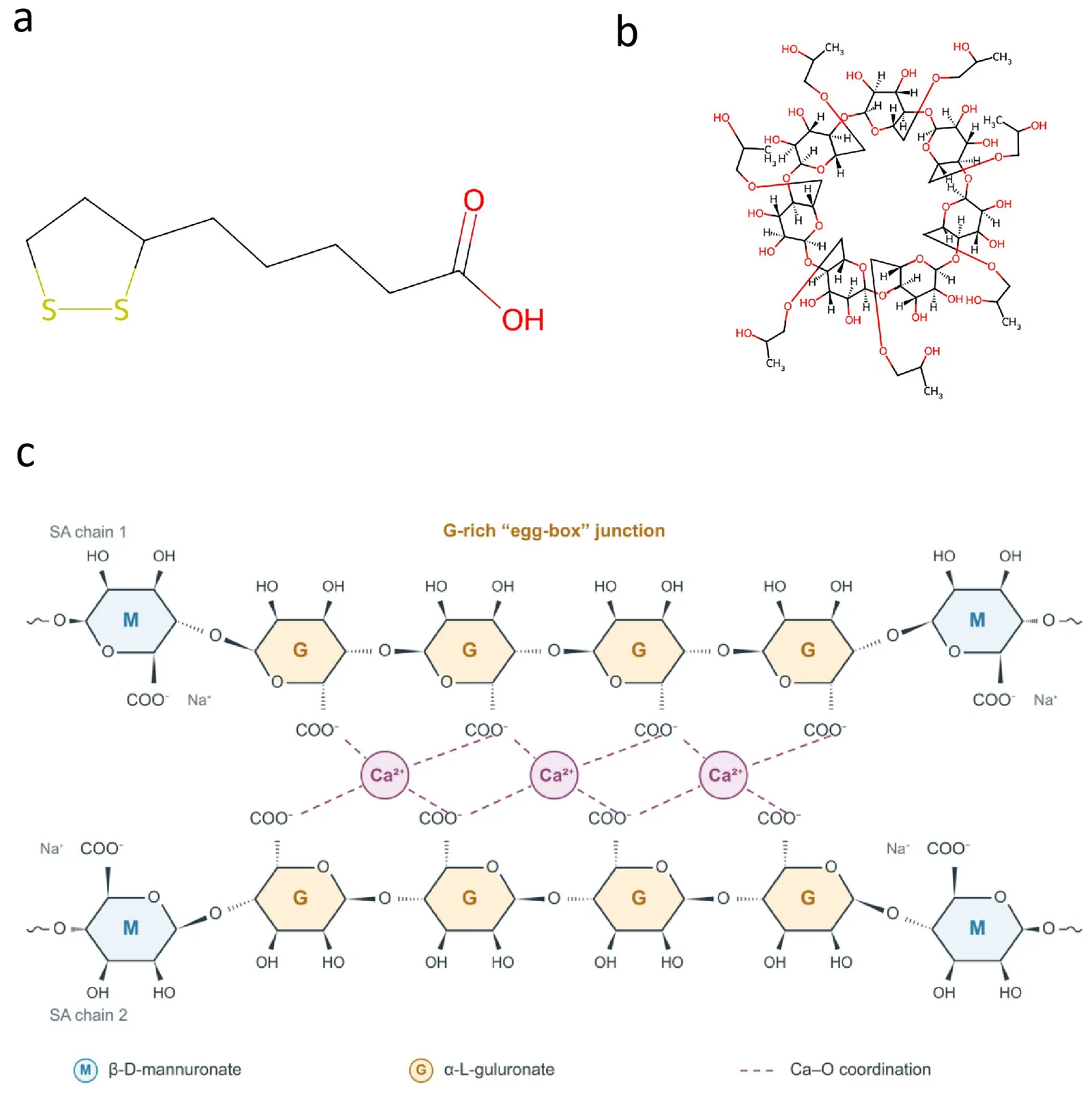
Chemical structures of Alpha-lipoic acid (ALA) and 2-hydroxypropyl-β-cyclodextrin (2-HP-β-CD) and schematic Ca^2+^-mediated cross-linking of sodium alginate (SA). (**a**) α-Lipoic acid (ALA). (**b**) Representative structure of 2-hydroxypropyl-β-cyclodextrin (2-HP-β-CD). (**c**) Two illustrative SA chain segments containing (1 → 4)-linked β-D-mannuronate (M, blue) and α-L-guluronate (G, orange) residues. Purple dashed lines indicate selected coordination contacts between Ca^2+^ and carboxylate oxygen atoms on adjacent chains, illustrating a G-rich “egg-box” junction.

An important reason for the low delivery efficiency of ALA lies in its low-pH instability and inadequate water solubility. Especially R-(+)-ALA is prone to degradation or polymerization under thermal and acidic conditions, thereby affecting its stability as an oral functional ingredient or active pharmaceutical ingredient [5]. ALA contains a 1,2-dithiolane ring and a carboxyl-terminated side chain, as shown in Figure 1a [6]. Previous studies have shown that R-ALA is sensitive to acid and heat, and improving its stability through molecular inclusion or carrier encapsulation is an important strategy to enhance the oral application potential of ALA [7]. Therefore, how to protect ALA in the gastric acidic environment, improve its aqueous dispersibility, and promote its release and absorption in the small intestine is a key scientific issue in the design of oral delivery systems for ALA.

Cyclodextrins (CDs) are a class of cyclic oligosaccharides with a “hydrophilic exterior, hydrophobic interior” cavity structure, which can encapsulate hydrophobic small molecules through host–guest interactions, thereby improving the apparent solubility, stability, and release behavior of drugs (Figure 1b) [8]. Studies on ALA have already demonstrated that cyclodextrin inclusion can improve the physicochemical properties of ALA [9]. Among various CD derivatives, 2-hydroxypropyl-β-cyclodextrin (2-HP-β-CD) is one of the most widely used due to its high aqueous solubility and favorable safety profile [10]. Research found that after forming a complex with γ-cyclodextrin, R-ALA exhibited enhanced stability under acidic and thermal conditions and was further used in the evaluation of oral bioavailability in humans. This study indicates that cyclodextrin inclusion not only serves as a solubilization method for ALA but also as an effective pretreatment strategy to improve its stability in the gastrointestinal environment [5].

However, simple cyclodextrin inclusion may still fail to achieve precise regulation of the release site and release rate of ALA. For oral delivery systems, an ideal carrier should not only improve the solubility and stability of hydrophobic active ingredients, but also reduce premature drug release in the acidic gastric environment and promote release in the neutral or weakly alkaline environment of the intestine [11]. Sodium alginate (SA) is a linear anionic polysaccharide composed of (1 → 4)-linked β-D-mannuronate (M) and α-L-guluronate (G) residues arranged in M^−^, G^−^, and alternating MG-blocks Ca^2+^ can coordinate with oxygen donors on adjacent alginate chains, promoting interchain association, particularly in G-rich regions. These junctions contribute to the formation of an ionically cross-linked gel network and are commonly described using the egg-box model. Figure 1c schematically illustrates the alginate chain structure and selected Ca^2+^-mediated interchain coordination contacts. Because calcium alginate gels shrink under low pH conditions, the electrostatic repulsion between molecular chains weakens, the gel network contracts, exhibiting low swelling, and thus limiting drug release [12]. Under higher pH conditions in the intestine, the carboxyl groups deprotonate to form negatively charged carboxylate groups, enhancing electrostatic repulsion between polymer chains, promoting swelling of the gel network, and facilitating drug diffusion and release [13]. Alginate-based materials are commonly used to construct pH-responsive oral delivery systems. Related studies have used calcium alginate hydrogel microspheres for gastrointestinal protection and intestinal targeted release of active substances such as drugs, proteins, polysaccharides, and probiotics, demonstrating their good acid protection and intestinal release potential [14,15].

This study therefore investigated a hierarchical delivery strategy that couples 2-HP-β-CD-mediated solubilization of ALA with release modulation by chitosan-coated calcium alginate microspheres [16]. At the molecular level, ALA/2-HP-β-CD inclusion complexes were prepared to increase the apparent aqueous solubility of ALA [17]. These inclusion complexes were introduced into the aqueous alginate phase and incorporated into a Ca^2+^-cross-linked network through emulsification/internal gelation, followed by chitosan coating. At the material level, this architecture was intended to limit premature release under gastric-stage conditions and permit greater release as the matrix hydrated and expanded under intestinal-stage conditions [18]. The central design hypothesis was that cyclodextrin-mediated solubilization increases the aqueous availability of ALA, while the surrounding polymer network modulates its outward transport. Phase-solubility, (X-ray diffraction) XRD, morphology, swelling, and release measurements were used to examine the relationships among these formulation levels.

## 2. Results

### 2.1. Liquid Chromatography–Mass Spectrometry (LC-MS) Analysis of Free ALA Following Acid Exposure

**Figure 2:**
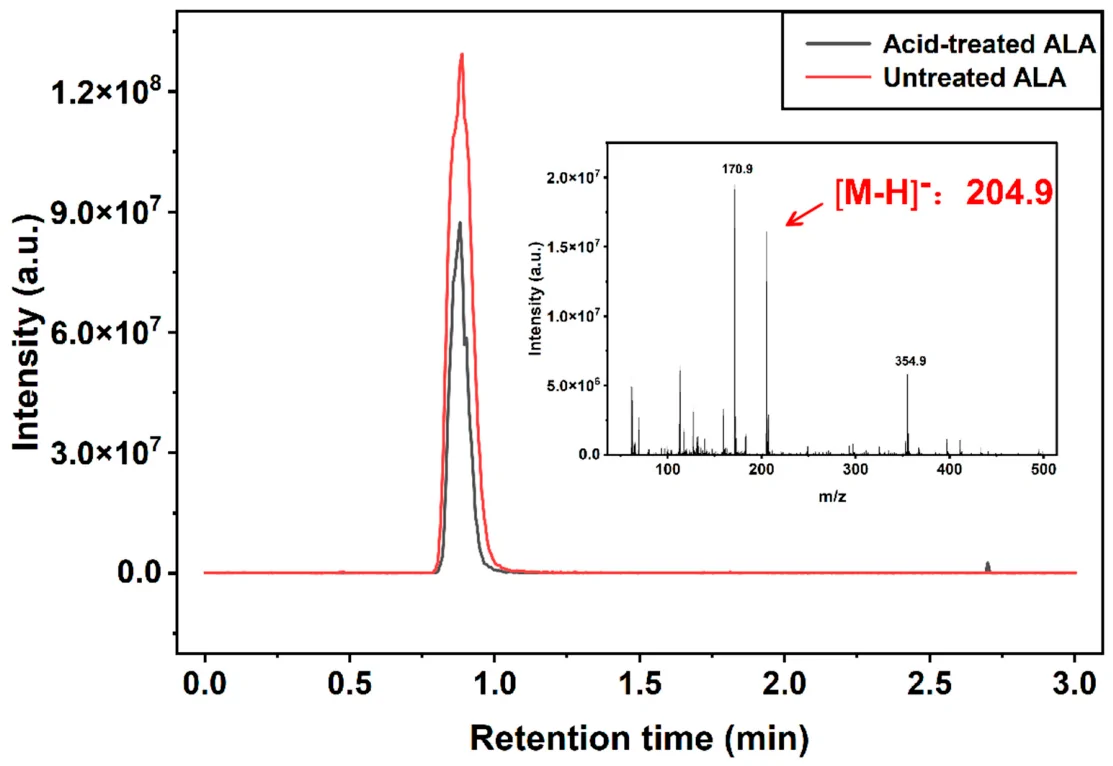
Liquid chromatography–mass spectrometry (LC-MS) analysis of ALA. Comparative LC-MS chromatograms before and after ALA treatment; Mass spectrum at 0.88 min, with *m*/*z* 204.9 corresponding to the [M − H]^−^ of ALA, and *m*/*z* 170.9 as its characteristic fragmentation.

To evaluate the stability of ALA in an acidic environment, this study employed LC-MS to analyze ALA samples before and after acid treatment (Figure 2). The untreated ALA sample exhibited a distinct characteristic chromatographic peak at approximately 0.88 min with [M − H]^−^ (204.9), with a sharp peak shape and high response intensity, indicating a high content of detectable free ALA in the sample. In comparison, the acid-treated ALA sample still showed a corresponding chromatographic peak at a similar retention time, but its peak area was significantly reduced and the peak response intensity decreased, suggesting a reduction in the detectable ALA content after acid treatment.

These results suggest a reduction in detectable monomeric ALA after acid treatment. This may partly reflect chemical changes in ALA under acidic conditions, potentially contributing to the observed decrease in chromatographic peak area. Previous studies provide support for this interpretation. Ikuta et al. reported reduced recovery of free R-ALA after incubation at pH 1.2 and 37 °C and discussed its susceptibility to polymerization under acidic conditions [5]. Uchida et al. also observed substantially lower residual ALA at pH 1.2 than at pH 3.0 or 6.8 [19]. Given that ALA must first be exposed to the gastric acid environment during oral administration, this result highlights the relevance of potential acid-associated chemical changes to the design of oral ALA delivery systems. The specific chemical changes involved, however, remain to be established. Therefore, the changes in LC-MS chromatographic peak areas before and after acid treatment provide an experimental basis for the subsequent construction of a protective delivery system. The enhancement of ALA dispersion through the formation of ALA/2-HP-β-CD inclusion complexes, combined with the use of chitosan-coated calcium alginate microspheres for pH-responsive release, may help limit premature release under gastric conditions and promote ALA release under simulated intestinal conditions.

### 2.2. Morphological Characterization of ALA/2-HP-β-CD Inclusion Complex-Loaded Alginate Microspheres

**Figure 3:**
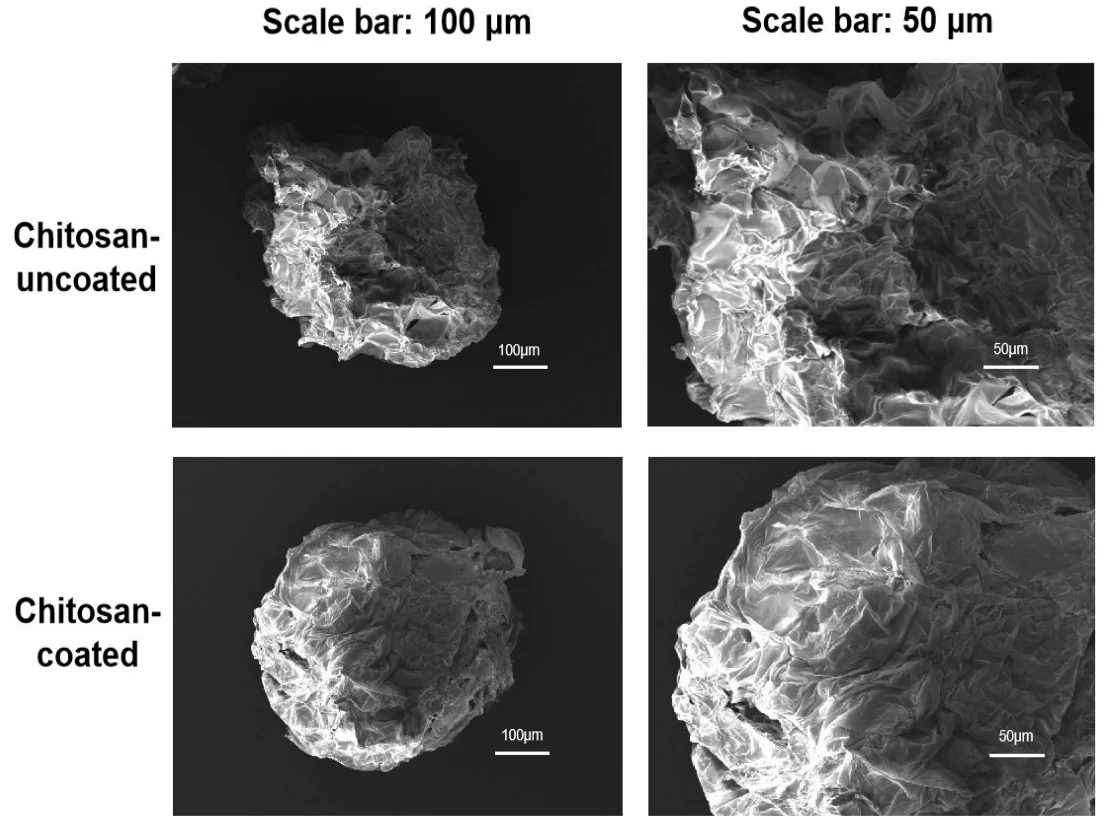
Morphology of chitosan-coated calcium alginate microspheres. Scanning electron microscopy (SEM) images of calcium alginate microspheres before and after chitosan coating. After coating with chitosan, the microsphere surface appears slightly smoother and more uniform, while still maintaining the overall microsphere morphology.

To improve the aqueous solubility of ALA and limit its premature release under gastric-stage conditions, ALA/2-HP-β-CD inclusion complexes were incorporated into chitosan-coated calcium alginate microspheres. The design rationale of this system is as follows: first, 2-HP-β-CD is used to form an inclusion complex with ALA to improve the aqueous solubility and dispersion state of ALA; subsequently, this inclusion complex is introduced into alginate microspheres, leveraging the pH-responsive properties of alginate materials to provide protection for ALA in the gastric acid environment and achieve release in the intestinal environment. Scanning electron microscope (SEM) observation results show that (Figure 3), the surface of microspheres without chitosan coating is slightly rough with tiny pores, which is a typical characteristic of Ca^2+^ cross-linked sodium alginate hydrogels [20]. After chitosan coating, the microsphere surface became slightly smoother and more uniform, indicating that the chitosan layer covered surface irregularities without significantly altering the overall size or spherical structure of the microspheres. The observed surface changes are consistent with modification of the microsphere surface following chitosan treatment. Previous studies provide a rationale for this coating strategy by showing that alginate–chitosan interactions can modify particle stability and release behavior [21,22]. These results, from a morphological perspective, demonstrate that ALA/2-HP-β-CD inclusion complexes can be effectively constructed as alginate microsphere carriers, providing a structural basis for subsequent oral delivery. The final chitosan-coated calcium alginate microspheres exhibited an ALA encapsulation efficiency of 76.91 ± 1.63% and a loading capacity of 18.74 ± 1.91% (weight/weight (*w*/*w*), dry-microsphere basis).

### 2.3. Phase Solubility Behavior of ALA with 2-HP-β-CD and Solid-State Characterization of ALA/2-HP-β-CD Inclusion Complex

**Figure 4:**
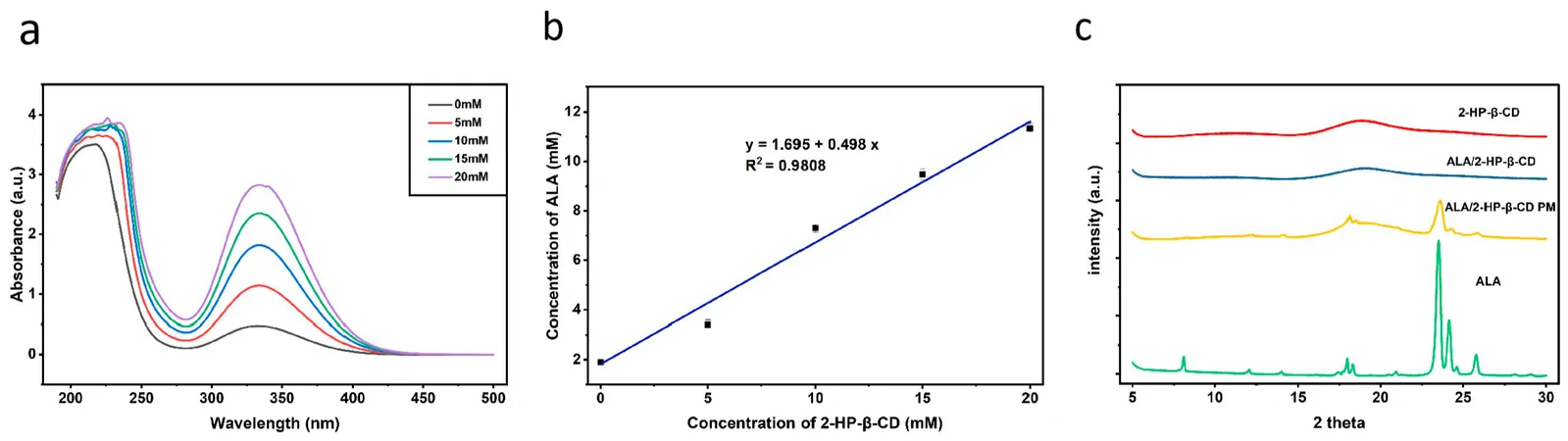
Physicochemical characterization of ALA and the ALA/2-HP-β-CD inclusion complex. (**a**) ultraviolet-visible (UV-Vis) full-wavelength scanning spectra of ALA in aqueous solutions of 2-HP-β-CD at different concentrations. (**b**) Phase solubility curve of ALA and 2-HP-β-CD. (**c**) X-ray diffraction (XRD) patterns of free ALA, free 2-HP-β-CD, inclusion complex, and the physical mixture (PM) of 2-HP-β-CD with ALA.

To investigate the solubilization effect of 2-HP-β-CD on ALA, Ultraviolet (UV) full-wavelength scanning was first conducted on ALA solutions at different concentrations of 2-HP-β-CD (Figure 4a). ALA exhibits a distinct characteristic absorption peak in the UV region. As the concentration of 2-HP-β-CD increased, the absorbance of ALA gradually increased, indicating an increase in the amount of ALA entering the aqueous phase system. These results indicate that 2-HP-β-CD can effectively increase the apparent aqueous solubility of ALA, which may be attributed to the host–guest inclusion complexation between the hydrophobic cavity of 2-HP-β-CD and the ALA molecule. The solubilization effect of 2-HP-β-CD was further quantitatively analyzed through phase solubility experiments (Figure 4b). Within the examined concentration range of 2-HP-β-CD, the apparent solubility of ALA increased linearly with the increase in 2-HP-β-CD concentration, and the phase solubility curve exhibited a typical type AL profile. This result is consistent with soluble complex formation between ALA and 2-HP-β-CD and is compatible with an apparent 1:1 complexation model. However, the stoichiometry cannot be established from the phase-solubility profile alone [23]. Over the investigated 2-HP-β-CD concentration range of 0–20 mM, linear regression yielded y = 1.695 + 0.498x (R^2^ = 0.9808), where x and y represent the concentrations of 2-HP-β-CD and dissolved ALA, respectively, both expressed in mM. The experimentally determined ALA solubility in the absence of 2-HP-β-CD was 1.900 ± 0.104 mM. Under an apparent 1:1 complexation model, the calculated complexation efficiency (CE) and apparent stability constant were 0.992 and 5.23 × 10^2^ M^−1^, respectively, at 303 K. Compared with free ALA, the addition of 2-HP-β-CD significantly improved the solubility of ALA in water, indicating that 2-HP-β-CD can serve as an effective solubilizing carrier for ALA. To further verify the formation of the ALA/2-HP-β-CD inclusion complex, this study employed XRD to perform solid-state characterization of free ALA, free 2-HP-β-CD, physical mixture, and inclusion complex (Figure 4c). The results showed that free ALA exhibited multiple sharp and intense characteristic diffraction peaks, indicating its distinct crystalline structure. In contrast, the diffraction peaks of 2-HP-β-CD were broad and diffuse, displaying typical amorphous features. In the physical mixture, characteristic crystal diffraction peaks of ALA could still be observed, indicating that simple physical mixing did not change the crystal state of ALA.

Notably, in the XRD pattern of the ALA/2-HP-β-CD inclusion complex, the characteristic diffraction peaks of ALA were significantly weakened or even disappeared, and the overall pattern approached the amorphous diffraction characteristics of 2-HP-β-CD. This change indicates a marked reduction in detectable crystalline ALA following complex preparation, consistent with the altered solid-state organization previously reported for ALA/HP-β-CD systems [23,24]. Therefore, the XRD results, considered together with the phase-solubility behavior and previous reports, support the formation of ALA/2-HP-β-CD inclusion complexes. In summary, the results of UV full-wavelength scanning, phase solubility experiments, and XRD collectively demonstrate enhanced apparent aqueous solubility of ALA and provide supporting evidence for inclusion complex formation with 2-HP-β-CD. The relevance of the inclusion complexes to the subsequent carrier design is that they increase the amount of ALA accessible to the aqueous phase, whereas the surrounding alginate–chitosan matrix is intended to modulate the transport of ALA-containing species into the release medium.

### 2.4. Particle Size Changes in ALA/2-HP-β-CD Inclusion Complex-Loaded Microspheres Under Simulated Gastrointestinal Conditions

**Figure 5:**
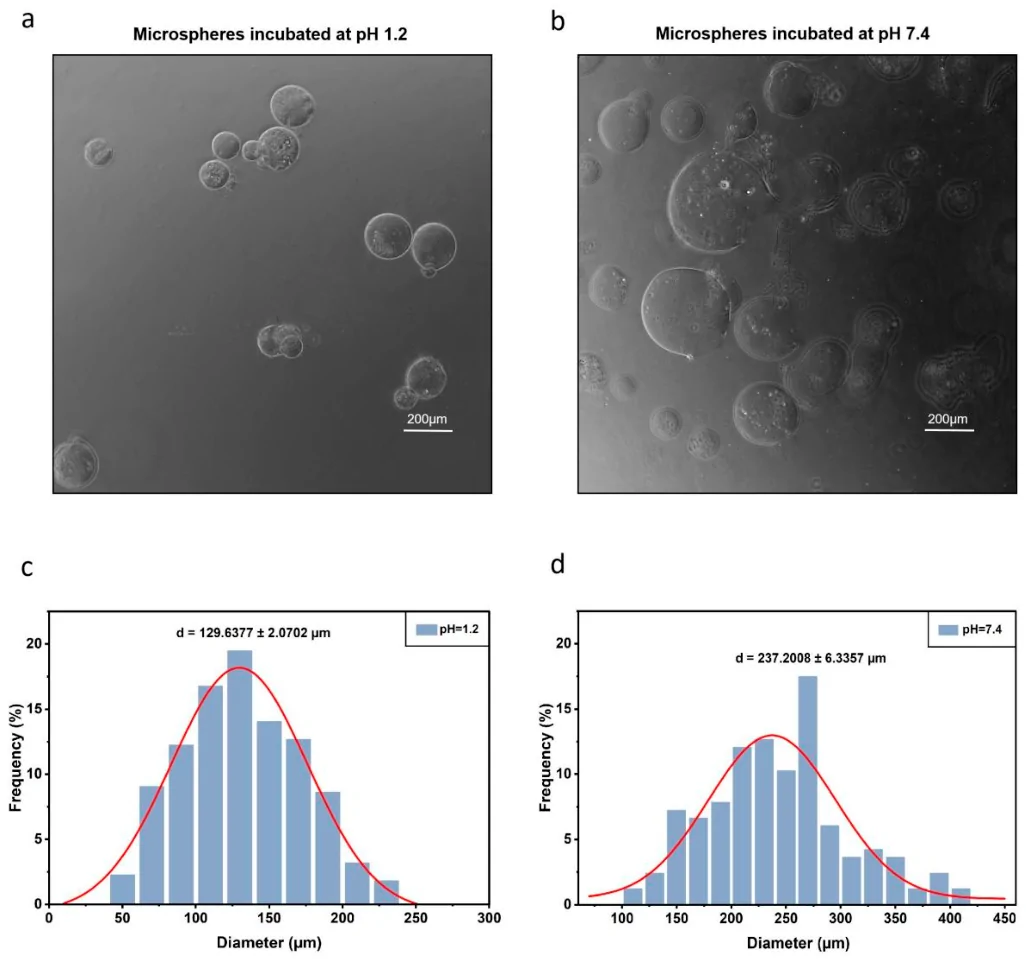
pH-responsive changes in the morphology and particle size of chitosan-coated calcium alginate microspheres loaded with the ALA/2-HP-β-CD formulation under simulated gastrointestinal conditions. (**a**,**b**) Optical microscopy images of microspheres after incubation in simulated gastric fluid (SGF, pH 1.2) and simulated intestinal fluid (SIF, pH 7.4), respectively. The microspheres exhibit a relatively dense morphology under acidic conditions, while showing significant swelling under intestinal pH conditions. (**c**,**d**) Quantitative analysis of microsphere particle size under different pH conditions.

The ALA/2-HP-β-CD inclusion complex was successfully incorporated into calcium alginate microspheres formed by Ca^2+^-mediated crosslinking of sodium alginate. The particle size and morphology of the microspheres in simulated gastric fluid (pH 1.2) and simulated intestinal fluid (pH 7.4) are shown in Figure 5. Under acidic conditions (pH 1.2), the non-lyophilized microspheres exhibited a smaller particle size with an average diameter of approximately 129.64 μm (Figure 5a,c), under neutral conditions (pH 7.4), the non-lyophilized microspheres swelled significantly, with an average diameter of approximately 237.20 μm (Figure 5b,d).

This pH-responsive swelling is consistent with literature reports: at low pH, protonation of the carboxyl groups in sodium alginate causes network contraction, reducing the volume of the microspheres; at high pH, deprotonation of carboxyl groups increases electrostatic repulsion and osmotic pressure, leading to microsphere swelling. These observations demonstrate a medium-dependent change in microsphere dimensions that is consistent with the proposed pH-responsive matrix behavior.

### 2.5. pH-Dependent Morphology and Swelling Behavior

**Figure 6:**
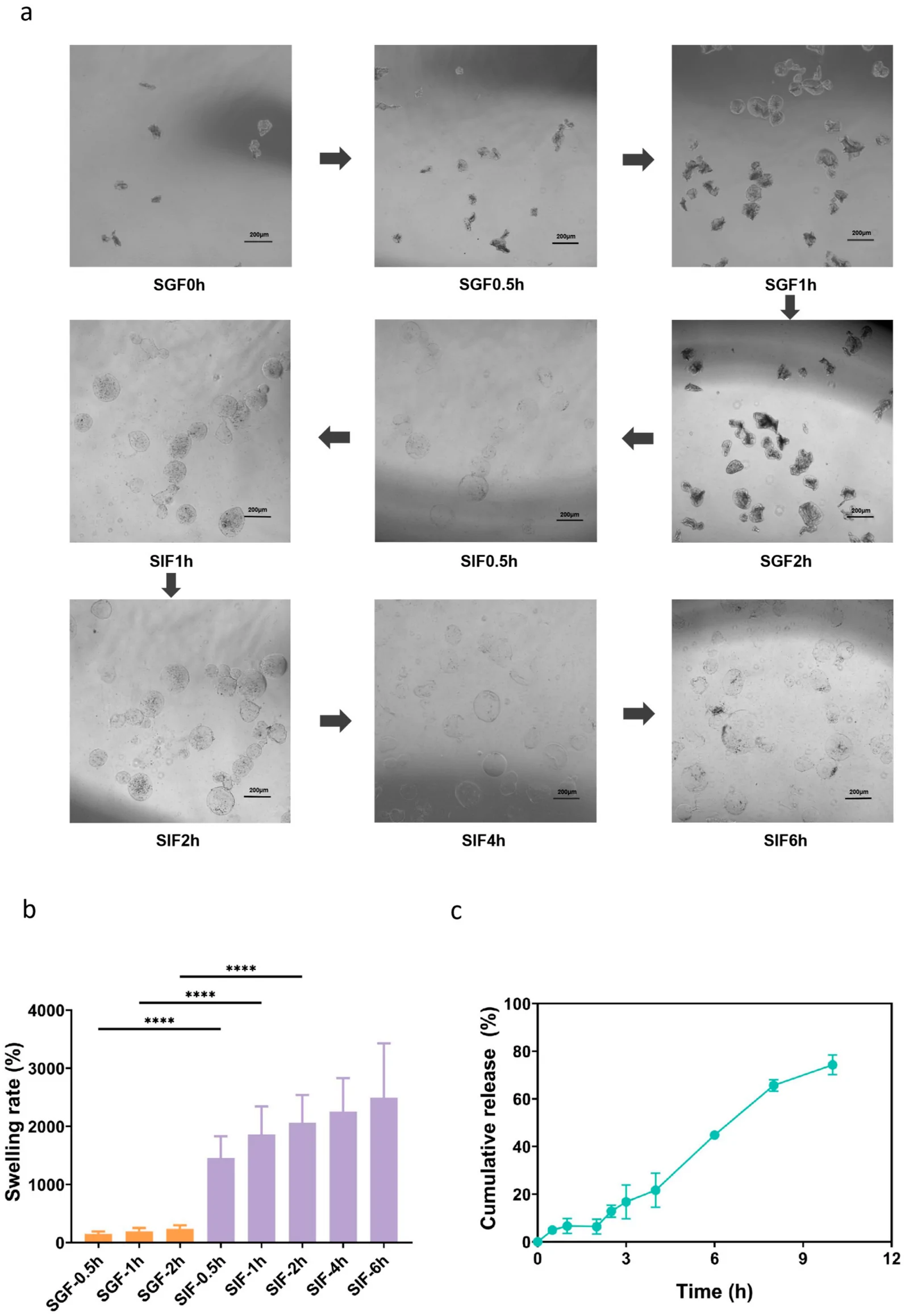
pH-dependent swelling behavior of ALA/2-HP-β-CD inclusion complex-loaded calcium alginate microspheres. (**a**) Optical images of microspheres during incubation in SGF and after addition of SIF to the SGF and adjustment of the mixture to pH 7.4. (**b**) Quantitative analysis of microsphere swelling behavior. The microspheres showed limited expansion in SGF and greater expansion in the pH-adjusted SGF/SIF mixture, with the largest measured swelling ratio at 6 h after the transition. Statistical differences between the indicated groups were analyzed using one-way analysis of variance (ANOVA) followed by Tukey’s post hoc multiple-comparisons test. **** (*p* < 0.0001). (**c**) In vitro apparent cumulative ALA-equivalent release profiles of ALA/2-HP-β-CD inclusion complex-loaded chitosan-coated calcium alginate microspheres.

To evaluate the pH-responsive morphology and swelling behavior of chitosan-coated calcium alginate microspheres containing ALA/2-HP-β-CD inclusion complexes, the freeze-dried microspheres were first incubated in simulated gastric fluid (SGF, pH 1.2) for 0, 0.5, 1, and 2 h. After 2 h, an equal volume of simulated intestinal fluid (SIF, pH 7.4) was added directly to the existing SGF, and the resulting 1:1 (volume per volume (*v*/*v*)) SGF/SIF mixture was adjusted to pH 7.4. Incubation was then continued for a further 0.5, 1, 2, 4, and 6 h under simulated intestinal-stage conditions. Optical microscopy showed that the microspheres remained contracted under gastric-stage conditions, and compared with the initial lyophilized state, the particle size did not show significant changes. Following the addition of SIF and adjustment of the mixed medium to pH 7.4, the microspheres exhibited pronounced swelling and appeared more spherical, with a smoother surface (Figure 6a). Quantitative particle-size analysis further supported this observation. The microsphere diameters remained relatively small and stable during the gastric stage but increased significantly under simulated intestinal-stage conditions, reaching their maximum after 6 h of intestinal-stage incubation. This pH-dependent swelling behavior is consistent with the characteristics of calcium alginate networks. Under acidic conditions, protonation of the carboxyl groups reduces electrostatic repulsion and promotes network contraction. After adjustment to pH 7.4, deprotonation of the carboxyl groups increases electrostatic repulsion and osmotic pressure, resulting in hydration and expansion of the polymer network. The swelling ratios are presented in Figure 6b. The greater expansion after the medium transition provides structural context for interpreting the release profile [25].

### 2.6. In Vitro Release Behavior and Release Kinetics

The in vitro apparent release profile of ALA-containing species, estimated by UV spectrophotometry at 334 nm after correction with matched blank release media, is shown in Figure 6c. During the initial 2 h incubation in simulated gastric fluid (SGF, pH 1.2), apparent ALA-equivalent release remained limited, with a cumulative release of 6.41 ± 3.13% at the end of the gastric stage. This limited release was consistent with the compact and contracted morphology of the microspheres observed under acidic conditions.

After SIF was added to the existing SGF and the mixed medium was adjusted to pH 7.4, the measured cumulative apparent ALA-equivalent release increased progressively, reaching 74.28 ± 4.14% after 10 h of total incubation. This increase occurred under conditions that also produced pronounced microsphere swelling. Matrix hydration and relaxation may facilitate outward transport of ALA-containing species, providing a link between the structural response and the release profile. Based on the estimated initial ALA amount of 18.74 mg, the cumulative release values of 6.41 ± 3.13% at 2 h and 74.28 ± 4.14% after 10 h correspond to nominal ALA-equivalent amounts of approximately 1.20 mg and 13.92 mg, respectively. These calculated amounts are derived from the apparent UV-based percentages and do not represent independently validated recovery of chemically intact ALA.

**Figure 7:**
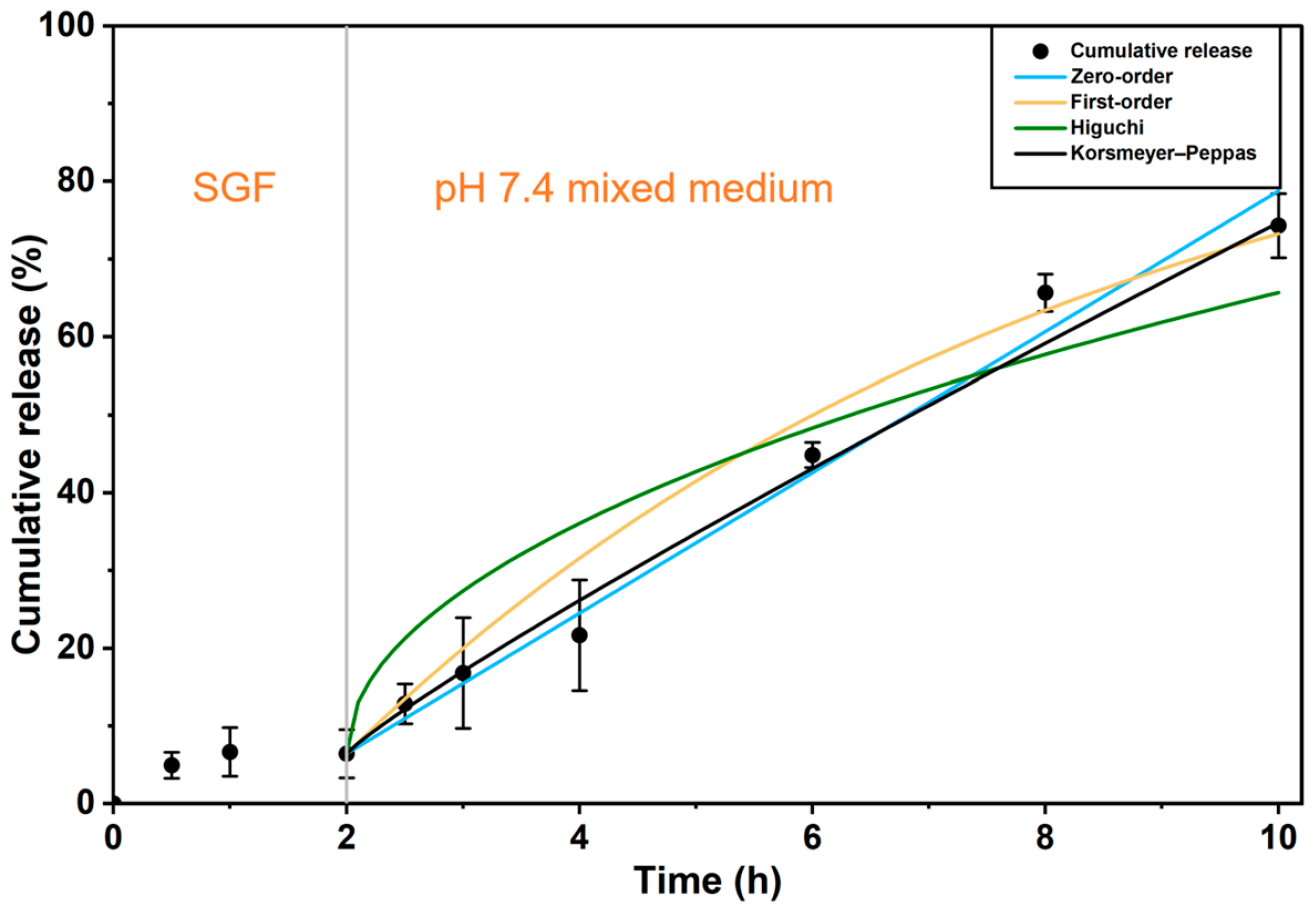
Experimental apparent cumulative ALA-equivalent release and kinetic model predictions for ALA/2-HP-β-CD-loaded chitosan-coated calcium alginate microspheres. Curves were calculated using the zero-order, first-order, Higuchi, and Korsmeyer–Peppas equations with the parameters reported in Table 1, taking the cumulative release measured at 2 h as the baseline.

Following the addition of SIF to the existing SGF and adjustment of the mixed medium to pH 7.4 at 2 h, release kinetics were analyzed using the cumulative release at 2 h as the baseline. Figure 7 presents the experimental release data together with predictions from the four kinetic models. As shown in Table 1, the baseline-corrected zero-order model provided the highest coefficient of determination, followed by the Korsmeyer–Peppas model. The Higuchi model showed comparatively lower fitting performance.

However, the observed release and swelling behavior suggests that drug release may be governed jointly by diffusion and the swelling/relaxation of the microsphere polymer network. These results indicate that apparent ALA release was approximately linear during the investigated pH 7.4 stage in the SGF/SIF mixed medium. The zero-order and Korsmeyer–Peppas models provided comparable empirical fits. However, these fits should not be interpreted as evidence of a unique release mechanism.

Overall, the limited release under gastric conditions followed by enhanced release under intestinal conditions supports the pH-responsive behavior of the delivery system. Together with the phase-solubility, XRD, morphological, and swelling results, the release profile is consistent with the proposed dual delivery strategy, in which 2-HP-β-CD improves the aqueous dispersibility of ALA, while the chitosan-coated calcium alginate network limits its release under acidic conditions and promotes release following exposure to intestinal pH.

## 3. Discussion

The delivery system was designed to couple the aqueous availability of ALA with its transport through a responsive polymer matrix. At the molecular level, ALA/2-HP-β-CD inclusion complexes provide a means of increasing the apparent amount of ALA accessible to the aqueous phase. Their incorporation into chitosan-coated calcium alginate microspheres introduces a surrounding network through which ALA-containing species must pass before entering the bulk release medium. The phase-solubility data support the solubilizing contribution of 2-HP-β-CD, whereas the swelling and release measurements characterize the complete coated microsphere system. Within the proposed design, ALA is the active payload, 2-HP-β-CD is the solubilizing host, calcium alginate forms the responsive matrix, and chitosan serves as a surface modifier [7,21,22].

The LC-MS results showed that the chromatographic response of detectable original ALA decreased markedly after acid treatment. Direct exposure of free ALA to strongly acidic conditions reduce the fraction remaining analytically available. This observation provides a relevant experimental rationale for protecting ALA during gastric transit. A recent comprehensive investigation of ALA-cyclodextrin systems also identified gastric instability as an important limitation and demonstrated that formulation with hydroxypropyl-β-cyclodextrin substantially improved the pharmaceutical performance of ALA under biorelevant conditions [24].

The linear increase in ALA solubility with increasing 2-HP-β-CD concentration and the AL-type phase-solubility profile are consistent with the formation of a soluble host–guest complex, compatible with an apparent 1:1 complexation model within the investigated concentration range. The weakening or disappearance of the characteristic ALA diffraction peaks in the inclusion-complex XRD pattern further indicates loss of long-range crystalline order and conversion of ALA into an amorphous or molecularly dispersed state. Both effects can reduce the crystal-lattice barrier to dissolution and thereby increase the concentration of ALA available in the aqueous phase. These findings agree closely with the recent work of Miljak et al., who identified HP-β-CD as an effective host for ALA and reported complete amorphization and rapid dissolution of spray-dried and lyophilized HP-β-CD-ALA complexes [24]. ALA/HP-β-CD inclusion systems with enhanced aqueous solubility and reduced crystallinity have also been reported [23]. However, XRD and phase-solubility analysis alone cannot fully establish the inclusion geometry or binding mode. Complementary differential scanning calorimetry, fourier transform infrared spectroscopy (FTIR), two-dimensional nuclear magnetic resonance (NMR), isothermal titration calorimetry, or molecular simulation would strengthen the structural interpretation.

Following incorporation of the inclusion complex into calcium alginate microspheres, chitosan coating produced a slightly smoother and more uniform surface while maintaining the overall microsphere structure. This observation is consistent with the formation of a polyelectrolyte layer through interactions between protonated amino groups of chitosan and carboxylate groups of alginates. Such a layer may reduce surface porosity, reinforce the ionically cross-linked alginate network, and restrict premature diffusion of the ALA/2-HP-β-CD complex. Meskelis et al. demonstrated that the sequence of alginate gelation and chitosan complexation influences particle structure and release behavior, supporting the use of a post-gelation coating strategy [21]. Zhao et al. directly compared coated and uncoated calcium alginate capsules and reported improved stability following chitosan coating under the conditions investigated [22]. Nevertheless, a systematic comparison of free ALA, ALA/2-HP-β-CD inclusion complexes, uncoated inclusion-complex-loaded alginate microspheres, and chitosan-coated inclusion-complex-loaded microspheres under matched gastric–intestinal conditions was not performed. The incremental contributions of complexation, alginate encapsulation, and chitosan coating to chemical preservation and release therefore remain unresolved. Future studies will compare these formulations at matched ALA doses under the same exposure conditions. These mechanistic interpretations are proposed explanations supported by the observed swelling and apparent release behavior and by the cited literature; the present experiments did not independently quantify the relative contributions of diffusion, polymer relaxation, matrix permeability, solubility, or complex dissociation.

The observed size changes demonstrate a structural response of the microspheres to the medium transition, providing a basis for discussing how matrix hydration may influence ALA transport. Under gastric conditions, protonation of alginate carboxyl groups reduces electrostatic repulsion between polymer chains and favors formation of a compact network with limited hydration. Under intestinal conditions, deprotonation increases electrostatic repulsion and osmotic water uptake, while the reduced protonation of chitosan may weaken alginate–chitosan ionic interactions. Matrix hydration and polymer-chain relaxation may increase the accessibility of aqueous diffusion pathways and alter matrix permeability, thereby facilitating the outward diffusion of dissolved ALA-containing species. Similar gastric contraction and intestinal swelling have been observed in recently reported alginate–chitosan and related pH-responsive hydrogel systems [26,27,28]. At the molecular level, 2-HP-β-CD-mediated solubilization can increase the amount of dissolved ALA available for transport within the hydrated matrix. The higher pH may also favor ALA ionization and aqueous solubility [24]. Reversible dissociation of ALA/2-HP-β-CD inclusion complexes may replenish unbound ALA as it diffuses into the release medium, while soluble complexes may also contribute to transport depending on their mobility within the network [29]. The observed release profile may therefore reflect the coupled effects of polymer relaxation, matrix permeability, diffusion, and ALA solubilization and complexation. The present measurements do not distinguish free from complexed ALA or resolve the relative contributions of these processes.

From a formulation perspective, the main contribution of this study is therefore not simply the preparation of another ALA carrier, but the integration of two complementary levels of control. The 2-HP-β-CD complex generates a more readily dispersible form of ALA, while the alginate–chitosan microsphere delays its exposure until the surrounding pH favors matrix hydration and release. Related studies have combined cyclodextrin inclusion with hydrogel encapsulation to improve both solubilization and controlled release of poorly water-soluble bioactive compounds [30]. The present system extends this concept to ALA and demonstrates pronounced gastric–intestinal release discrimination. Its preparation from natural polysaccharides and ionically cross-linked materials may also offer advantages for oral multiparticulate formulation. However, evidence of improved solubility and intestinal release cannot yet be equated with improved oral bioavailability, because epithelial permeability, intestinal residence, first-pass metabolism, and interactions with gastrointestinal contents remain unresolved [7,24]. Hydrodynamic size, polydispersity index (PDI), and zeta potential before and after release in intestinal media were not measured. Optical microscopy assessed hydrated microspheres, whereas SEM examined freeze-dried microspheres. Future dynamic light scattering (DLS) and zeta-potential measurements should examine suitable dispersed particles in the release medium, if present. Comparisons with SEM should consider differences in particle type and hydration state.

Of course, this study still has certain limitations. First, the current experiments primarily relied on in vitro acid treatment, phase solubility studies, X-ray diffraction (XRD) analysis, morphological observation, and evaluation of swelling and release behavior, without directly assessing intestinal absorption, systemic bioavailability, or therapeutic benefit. Accordingly, the present findings support improvements in formulation-level physicochemical properties and characterize pH-responsive apparent release under simplified gastrointestinal conditions; the potential benefits for oral delivery require further biological evaluation. Second, in vitro simulation of gastric and intestinal fluids cannot fully replicate the actual gastrointestinal environment [31], which also involves factors such as gastric emptying, intestinal peristalsis, bile salts, digestive enzymes, and the mucosal layer. Third, chemical preservation of ALA during gastric and intestinal exposure was not directly established by the UV-based release assay. The release values were estimated using an ethanol-based calibration with matched release-medium blank correction; equivalence of the analytical response between ethanol and the aqueous release media was not independently verified. Accordingly, the 334 nm signal is interpreted as an apparent response from ALA-containing species and does not by itself distinguish free ALA from ALA associated with 2-HP-β-CD or establish the chemical integrity of released ALA. Future analytical validation will include calibration and spike-recovery experiments in the relevant release media, together with independent chromatographic quantification. Therefore, the reported release percentages and kinetic parameters are interpreted as apparent UV-derived ALA-equivalent values and empirical descriptors of the observed release profile. The present results characterize in vitro pH-responsive release, while protection against acid-associated chemical changes remains a proposed benefit. Future work will assess intact ALA in the release medium and recovered microspheres after both stages using chromatographic separation and mass spectrometric identification, with comparison to unencapsulated ALA, and will evaluate the retained antioxidant activity. Therefore, future research should include in vivo pharmacokinetic studies [9,32], gastrointestinal distribution assays, and functional efficacy evaluations to clarify the actual impact of this delivery system on improving the oral bioavailability and biological effects of ALA [33,34].

In conclusion, the present study demonstrates increased apparent ALA solubility associated with ALA/2-HP-β-CD complexation, together with pH-dependent swelling and apparent release from the chitosan-coated calcium alginate microspheres in vitro. 2-HP-β-CD primarily contributes to the apparent solubilization of ALA, while the calcium alginate microspheres were associated with limited apparent release under acidic conditions and increased apparent release after the intestinal-stage transition [35]. This system may provide a potential approach for further investigating intestinal-stage delivery of ALA and may serve as a reference for developing oral delivery carriers for other active ingredients that are unstable or poorly water-soluble under acidic conditions. Further intestinal transport, pharmacokinetic, and pharmacodynamic studies are required to determine whether these formulation properties translate into improved intestinal absorption, systemic bioavailability, or therapeutic benefit [36].

## 4. Materials and Methods

### 4.1. Materials

The reagents used in the experiments were purchased as follows: ALA from Shanghai Yuanye Bio-Technology Co., Ltd. (Shanghai, China), 2-HP-β-CD (purity ≥ 97%), sodium alginate, chitosan, calcium carbonate from Aladdin Reagent Co., Ltd. (Shanghai, China), Span 80 (sorbitan monooleate) from Sigma-Aldrich (Shanghai, China), ammonium acetate (chromatographic grade) from Macklin Biochemical Co., Ltd. (Shanghai, China). All other chemicals were of analytical reagent grade. Ultrapure water (1.82 MΩ·m) was prepared using a Thermo Scientific ultrapure water system (Thermo Fisher Scientific Inc., Waltham, MA, USA) and used for all experiments.

### 4.2. Acid Treatment of ALA

ALA was initially dissolved in ethanol at a concentration of 1 mg/mL for both the control and acid-treatment groups. Water was added to the control group, whereas 0.1 M HCl was added to the acid-treatment group to adjust the mixture to pH 1.2. Both groups were incubated at 37 °C for 2 h. Following incubation, both samples were adjusted to pH 7 using 0.1 M NaOH. The final sample volume and ethanol proportion were identical between the two groups. Both samples were centrifuged at 12,000× *g* for 10 min to remove any residual particulate material, and the supernatants were subjected to LC-MS analysis. LC-MS analysis was performed using the negative ion scanning mode of Agilent 1290II (Agilent Technologies, Inc., Santa Clara, CA, USA)-AB SCIEX 5500+.

### 4.3. Preparation of 2-HP-β-CD/ALA Inclusion Complex

The preparation of the inclusion complex followed the method reported in the literature with slight modifications [23]. First, 2-HP-β-CD was dissolved in distilled water at a concentration of 15 mM. Excess ALA was added to the dissolved 2-HP-β-CD solution, and the mixture was shaken in a constant temperature shaker at 303 K for 72 h until the system reached equilibrium. The supernatant was filtered through a 0.45 μm polyethersulfone (PES) microfiltration membrane. The resulting solution was frozen at −80 °C overnight and then lyophilized in a freeze dryer for 48 h to obtain the 2-HP-β-CD/ALA lyophilized powder.

### 4.4. Phase Solubility Study

The phase solubility analysis of ALA in 2-HP-β-CD solutions was performed using the method reported [37]. An excess amount of ALA (100 mg) was added into aqueous solutions of 2-HP-β-CD at various concentrations (0–20 mM). The premixed solutions were subjected to ultrasonic treatment for 30 min and then shaken on a constant temperature oscillator at 303 K for 72 h. After the system reached equilibrium, 2 mL of each solution was filtered through a 0.45 μm PES microfiltration membrane. After appropriate dilution, the filtrate was measured using a ultraviolet–visible (UV-Vis) spectrophotometer (METASH) at a wavelength of 334 nm. The concentration of ALA in the solution was calculated based on the standard curve. The phase solubility curve was plotted with the concentration of 2-HP-β-CD on the x-axis and the concentration of ALA on the y-axis. CE was calculated from the slope of the linear phase-solubility plot using CE = slope/(1 − slope), with both ALA and 2-HP-β-CD concentrations expressed in molar units. This calculation was based on an apparent 1:1 complexation model. The apparent stability constant was calculated as K_app_ = slope/[S_0_(1 − slope)], where S_0_ is the measured equilibrium solubility of ALA in the absence of 2-HP-β-CD, expressed in mol/L [38].

### 4.5. XRD and SEM Characterization

X-ray diffraction (XRD) patterns of ALA, 2-HP-β-CD, the physical mixture of 2-HP-β-CD and ALA, the ALA/2-HP-β-CD inclusion complex, and various substances constituting the hydrogel were measured using an XRD instrument (Rigaku Smartlab, Rigaku Corporation, Akishima, Tokyo, Japan). The XRD data acquisition parameters were: tube voltage 40 kV, tube current 40 mA, diffraction angle 2θ scanning range 5–80°, and scanning rate 5°/min.

Scanning electron microscopy (SEM, Regulus 8100, Hitachi High-Tech Corporation, Tokyo, Japan) was used to observe the surface microstructure of freeze-dried gel microspheres with or without chitosan coating. The samples were coated with gold powder before observation.

### 4.6. Preparation of Chitosan-Coated Alginate Microspheres Loaded with ALA/2-HP-β-CD Inclusion Complexes

The preparation of hydrogel microspheres employed an emulsification/internal gelation method [39] with modifications to produce microspheres containing ALA/2-HP-β-CD. First, 10 mL of ALA/2-HP-β-CD was mixed with 20 mL of 3.0% (weight per volume (*w*/*v*)) sodium alginate solution to obtain a 2.0% (*w*/*v*) sodium alginate solution. Micronized calcium carbonate (calcium to sodium alginate mass ratio of 7.3%) was added to the mixture. After homogenization, the mixture was dispersed into soybean oil containing 1% (*v*/*v*) Span 80 (internal phase ratio of 50%, *v*/*v*) using a mixer (RCH1000) with stirring at 300 rpm. After 30 min of emulsification, 20 mL of soybean oil containing glacial acetic acid (acid/CaCO_3_ molar ratio of 3.5) was added to the water-in-oil emulsion, and crosslinking was continued for 30 min. Acetic acid promoted the dissolution of CaCO_3_ within the alginate-containing aqueous droplets, releasing Ca^2+^ to induce internal ionic gelation. After spherification, the mixture was centrifuged at 3000× *g* for 10 min to remove the oil phase, and the microspheres were recovered by washing twice with acetate buffer at pH 5.5. Chitosan coating was performed following the method reported by Liu and Krishnan et al. [40] with slight modifications; 0.2 g of chitosan was dissolved in 90 mL of distilled water, and 0.4 mL of glacial acetic acid was added for acidification to achieve a final concentration of 0.2% (*w*/*v*). Subsequently, the pH was adjusted to between 5.7 and 6.0 by adding 1 M sodium hydroxide. The washed microspheres were immersed in 100 mL of chitosan solution and shaken at 100 rpm for 20 min. After coating, the microspheres were rinsed with distilled water, filtered, and stored in acetate buffer at 4 °C.

### 4.7. Determination of Encapsulation Efficiency and Loading Capacity

The drug-loading performance of the microspheres was evaluated in terms of encapsulation efficiency (EE) and loading capacity (LC) [41]. After chitosan coating and washing, the microspheres were lyophilized, and the total dry mass recovered from each batch was recorded. An accurately weighed, representative aliquot (10 mg) was suspended in 0.2 M trisodium citrate solution (pH 7.4) for 1 h to disrupt the calcium alginate network and extract The ALA concentration in the citrate extract was estimated by UV-Vis spectrophotometry at 334 nm using the same calibration curve prepared from ALA standards dissolved in absolute ethanol. The same ethanol-based calibration curve was used for the ALA input, encapsulation efficiency, loading capacity, and release measurements. Drug-free microspheres containing the carrier components and 2-HP-β-CD were processed in parallel to assess background interference. The mass of ALA in the aliquot was calculated from its measured concentration, final extract volume, and dilution factor. The total ALA mass retained in each batch was estimated by multiplying the measured ALA content per unit dry mass by the total dry mass of the recovered microspheres. EE and LC were calculated as follows:

$$\mathrm{EE}(\%)=\frac{M_{1}}{M_{0}}\times 100$$

$$\mathrm{LC}(\%)=\frac{M_{1}}{M_{2}}\times 100$$

where M_0_ is the mass of ALA introduced into the microsphere preparation, M_1_ is the total mass of ALA retained in the final microspheres recovered from the same batch, and M_2_ is the total dry mass of those microspheres. M_0_ was determined from the measured ALA concentration and the actual volume of the ALA/2-HP-β-CD formulation added before emulsification. Measurements were performed on 3 independently prepared batches, and the results are expressed as mean ± standard deviation. This procedure provides a consistent ALA-equivalent assay scale but does not independently establish identical ALA response or recovery in the citrate extract and the aqueous release media.

### 4.8. Assessment of pH-Dependent Morphology and Swelling

Freeze-dried microspheres (10 mg) were incubated statically at 37 °C in 1 mL of gastric-stage medium, prepared from 0.1 M HCl and adjusted to pH 1.2, for 2 h. Without removing the microspheres or the gastric-stage medium, 1 mL of SIF was added. The resulting mixture was adjusted to pH 7.4 with NaOH (0.1 M), and incubation continued statically at 37 °C for a further 6 h. Micrographs were acquired before incubation and at 0.5, 1, and 2 h of the gastric stage and 0.5, 1, 2, 4, and 6 h after the medium transition. Microsphere diameters were measured from calibrated images using ImageJ version 1.54p. Assuming an approximately spherical shape, an apparent volume was estimated for each measured microsphere as V = πd^3^/6, where d is its measured diameter. Where V_0_ is the initial volume of the lyophilized microspheres, and Vt is the volume after immersion for a specific time, swelling ratio (%) = [(V_t_ − V_0_)/V_0_] × 100.

### 4.9. In Vitro Release Study

First, 100 mg of dried chitosan-coated alginate hydrogel microspheres was incubated in 10 mL of SGF, and incubated in a water bath shaker (ZQZY-AR8) at 37 °C and 100 rpm for 2 h. Based on the mean ALA loading capacity of 18.74% (*w*/*w*, dry-microsphere basis; Section 2.2), the initial ALA content of this 100 mg microsphere sample was estimated to be 18.74 mg. This estimated amount was used as the denominator for calculating the cumulative apparent release percentage. Samples of 1 mL were taken at 0.5 h, 1 h, and 2 h, respectively, and an equal volume of fresh SGF was added. Subsequently, the solution was mixed with SIF at a ratio of 1:1 (*v*/*v*), the pH was adjusted to 7.4, and incubation was continued in a water bath shaker (37 °C, 100 rpm). At time points of 0.5 h, 1 h, 2 h, 4 h, 6 h and 8 h, 1 mL of sample was taken and supplemented with an equal volume of fresh SIF. Following filtration through a 0.45 μm membrane, the release samples were analyzed by UV spectrophotometry at 334 nm. A calibration curve was established using ALA standards prepared in absolute ethanol over a concentration range of 0–1 mg/mL, with absolute ethanol serving as the calibration blank. The same ethanol-based calibration curve was used for all ALA-related measurements in this study. The common calibration conversion factor was therefore applied consistently in the release-percentage calculation. Unfortunately, this procedure did not independently verify equivalent molar absorptivity or recovery of ALA in the loading extract, SGF, and the pH-adjusted SGF/SIF medium. For background correction, blank chitosan-coated calcium alginate microspheres prepared without ALA/2-HP-β-CD inclusion complexes were subjected to the same release protocol. Blank release media were collected at the corresponding release stages and sampling times and filtered in the same manner as the loaded-microsphere samples. Their absorbances were used to correct the sample readings before apparent ALA concentrations were estimated from the calibration curve. UV-Vis wavelength scans showed a characteristic absorption band of ALA around 334 nm, whereas 2-HP-β-CD exhibited no distinct absorption peak at this wavelength under the tested conditions, supporting the selection of 334 nm for ALA quantification (Figure S1).

### 4.10. Release Kinetics

To further describe the apparent release profile of ALA from the microspheres, the in vitro cumulative release data were fitted to commonly used kinetic models. Because SIF was added to the existing SGF and the resulting mixture was adjusted to pH 7.4 at 2 h, kinetic modeling was performed for the post-transition stage rather than across the entire release profile. The intestinal-stage time was defined as T = t − 2 h, and Q_0_ was the cumulative release measured at the end of the SGF stage. The following baseline-corrected equations were used:

zero-order: Q_T_ − Q_0_ = k_0_T,

first-order: ln[(100 − Q_T_)/(100 − Q_0_)] = −k_1_T,

Higuchi: Q_T_ − Q_0_ = k_H_T^1/2^,

Korsmeyer–Peppas: lnF_T_ = lnk_KP_ + nlnT.

Here, t denotes the total incubation time in hours. Q_T_ is the cumulative apparent ALA-equivalent release (%). The k denotes the model-specific release constant. F_T_ is the dimensionless, baseline-corrected normalized release fraction, and n is the release exponent. All four models were fitted to the mean release data at total incubation times of 2.5, 3, 4, 6, 8, and 10 h. The model parameters were obtained by linear regression of the corresponding transformed variables. For cross-model comparison, R^2^ was calculated on the original cumulative-release scale. The fitted parameters are empirical descriptors of the apparent release profile under the present assay conditions.

### 4.11. Statistical Analysis

All experiments were performed at least in triplicate unless otherwise stated. Data are presented as mean ± standard deviation (SD). Statistical analysis and graph plotting were performed using GraphPad Prism version 10.4.1 or OriginPro 2026b version 10.3.5.222 software. Comparisons between two independent groups were performed using an unpaired two-tailed Student’s *t*-test. Comparisons among more than two groups were performed using one-way analysis of variance (ANOVA) followed by Tukey’s post hoc multiple-comparisons test. A two-sided *p* < 0.05 was considered statistically significant. Statistical significance is indicated as follows: * *p* < 0.05, ** *p* < 0.01, *** *p* < 0.001, and **** *p* < 0.0001. The exact statistical test, sample size, and error definition are provided in the corresponding figure legends.

## 5. Conclusions

This study developed chitosan-coated calcium alginate microspheres loaded with ALA/2-HP-β-CD inclusion complexes to improve apparent aqueous solubility and achieve pH-responsive release in vitro. The 2-HP-β-CD inclusion complex reduced the crystalline characteristics of ALA and enhanced its solubility, while the resulting chitosan-coated calcium alginate microspheres exhibited limited apparent ALA release under simulated gastric conditions and increased apparent release following the transition to intestinal-stage conditions. This hierarchical delivery system therefore provides a promising strategy for improving the oral delivery of ALA and may also be applicable to other poorly water-soluble and acid-sensitive bioactive compounds. These physicochemical and in vitro release findings do not establish enhanced intestinal absorption, systemic bioavailability, or therapeutic benefit, which require further biological evaluation.

## Figures and Tables

**Table 1:** Release kinetics parameters of chitosan-coated calcium alginate microspheres loaded with ALA/2-HP-β-CD complexes.

Model	Linear Equation Used for Fitting	k	R^2^
Zero-order	Q_T_ − Q_0_ = k_0_T	k_0_ = 9.042% h^−1^	0.986
First-order	$\ln\left[\left(100-Q_{T}\right)/\left(100-Q_{0}\right)\right]=-k_{1}T$	k_1_ = 0.1565 h^−1^	0.968
Higuchi	Q_T_ − Q_0_ = k_H_T^1/2^	k_H_ = 20.953% h^−1/2^	0.878
Korsmeyer-Peppas	lnF_T_ = lnk_KP_ + nlnT	k_KP_ = 0.1133 h^−n^	0.985

Note: The cumulative release data were fitted to zero-order, first-order, Higuchi, and Korsmeyer-Peppas models, respectively, where k denotes the release rate constant and R^2^ represents the goodness of fit. For the Korsmeyer–Peppas model, n is the release exponent (n = 0.895). The fitted parameters are empirical descriptors of the apparent release profile and are not interpreted as validated intrinsic release-rate constants.

## Data Availability

The original contributions presented in this study are included in the article and Supplementary Materials. Further inquiries can be directed to the corresponding authors.

## Supplementary Materials

The following supporting information can be downloaded at https://www.mdpi.com/article/10.3390/ijms27208998/s1.
