# Supplementary material for: Chitosan-Coated Calcium Alginate Microspheres Loaded with Alpha-Lipoic Acid/2-HP-β-CD Inclusion Complexes Enhance Aqueous Solubility and Enable pH-Responsive Intestinal Release
Source: Int J Mol Sci. 2026 Oct 11;27(20):8998. doi: 10.3390/ijms27208998 (PMC13705165; doi:10.3390/ijms27208998)
Supplement: Supplementary file 1 [file ijms-27-08998-s001.zip › ijms-4573737-supplementary.pdf]

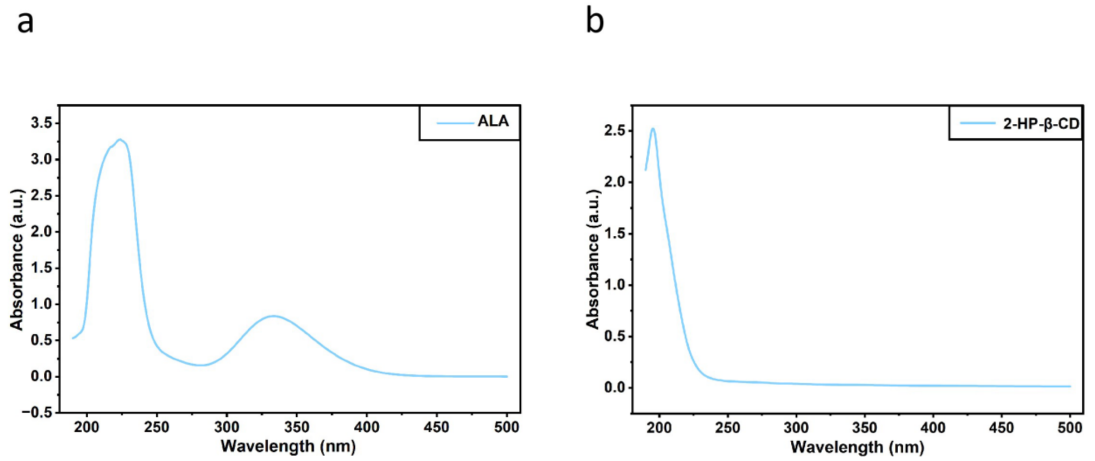

Figure S1. UV-Vis absorption spectra of (a) ALA and (b) 2-HP-β-CD. ALA exhibited a characteristic absorption band around 334 nm, whereas no distinct absorption peak of 2-HP-β-CD was observed at this wavelength under the tested conditions.
